# Bacterial pore-forming toxins: mechanisms and implications for host immunity

**DOI:** 10.1042/BSR20250103

**Published:** 2026-03-23

**Authors:** Kusum Lata, Shraddha Gandhi, Shamaita Chatterjee, Sindhoora Puravankara, Aakanksha Chauhan, Koyel Nandy, Kasturi Sarkar, Geetika, Gurvinder Kaur, Mandeep, Kausik Chattopadhyay

**Affiliations:** Department of Biological Sciences, Indian Institute of Science Education and Research Mohali, Sector 81, SAS Nagar, Manauli, Mohali, Punjab 140306, India

**Keywords:** Bacterial pore-forming toxins (PFTs), Cell signalling and cell death, host-pathogen interactions

## Abstract

Pore-forming toxins (PFTs) are an ancient class of protein toxins specialized in membrane disruption and aiding in the pathogenicity of several different pathogens. These versatile toxins are multifunctional virulence factors that manipulate host signaling and immune responses and modulate the cellular fate. The pore-formation mechanism of PFTs proceeds in a stepwise manner, initiated by receptor binding, followed by oligomerization and membrane insertion. Beyond membrane disruption, PFTs trigger a cascade of immune signaling, inflammasome activation, and diverse cell death pathways such as apoptosis, pyroptosis, necroptosis, and ferroptosis. Additionally, there is increasing evidence suggesting that many PFTs undergo endocytosis and traffic to organelles such as mitochondria, lysosomes, the endoplasmic reticulum, and Golgi, where they modulate intracellular functions. Interestingly, some functions of PFTs are also independent of pore formation, highlighting the functional versatility of PFTs. Technological advancements ranging from cryo-electron microscopy and high-speed AFM to AI-guided modeling, single-molecule imaging, and membrane-mimetic systems have been central in providing structural and mechanistic insights into PFT biology. There has been the discovery of new toxins as well as new toxin families; many of them are antibacterial PFTs deployed in the microbial competition. The growing insights into PFT biology have opened new avenues for therapeutic innovation, both by developing strategies to neutralize PFT-mediated pathogenesis and by engineering PFTs for vaccine development and cancer treatment. In this review, we provide a comprehensive overview of PFT biology within the broader context of host–pathogen interactions and highlight the key structural, mechanistic, and cellular questions that remain unresolved.

## Pore-forming toxins: evolutionary tools for disruption and subversion

The intricate network of host–pathogen interactions presents a fascinating landscape of an arms race that involves co-evolving strategies, counter-strategies, and environmental pressures. Such dynamic interactions generally are strong drivers for the evolution of distinct molecular tactics, giving rise to a diverse arsenal of virulence factors. Among these, pore-forming toxins (PFTs) stand out as one of the most widespread and multifunctional classes of bacterial proteins [1,2]. These ancient and versatile molecules are found across nearly all kingdoms of life, where they are weaponized in attack or defense systems. PFTs contribute to a range of life-threatening food-borne infections, including listeriosis, gas gangrene, and necrotizing pneumonia [3,4]. These proteins are very strategically specialized molecular innovations secreted not just to kill the target cells but also to manipulate the host cellular processes and promote microbial survival [5,6]. The ability of PFTs to be cytolytic and regulatory at the same time serves as both a challenge and an opportunity in biomedical research. Understanding how such protein toxins operate at the molecular level provides critical insight into the microbial pathogenesis as well as host defense mechanisms.

PFTs are primarily specialized in inserting transmembrane pores within the cellular membranes, which is the most critical barrier protecting the cellular and organellar integrity. Based on the secondary structure of membrane-spanning motifs of the transmembrane pores, PFTs are broadly categorized into classes of α-PFTs (with membrane-spanning α-helices) and β-PFTs (with membrane-spanning β-barrels) [4,7]. Despite having a common outcome of pore formation, PFTs have diversified in their mechanism of membrane recognition, assembly, and activation. There are more than 80 families of PFTs, classified based on structure, function, and mechanism [8]. The major families of α-PFTs include the colicins, actinoporins (PFTs from sea anemones), cytolysin A family, and RTX toxins. In contrast, the principal families of β-PFTs are the hemolysin family, aerolysin family, cholesterol-dependent cytolysins (CDCs), membrane attack complex (MACPF)/perforin superfamily, and leukocidin family [4,9]. There are numerous members in each family of PFTs with established pathophysiological significance in various disease conditions, where they exert their toxic effects by triggering colloid-osmotic lysis, hijacking the immune system and modulating the host signaling pathways [2].

**Figure 1:**
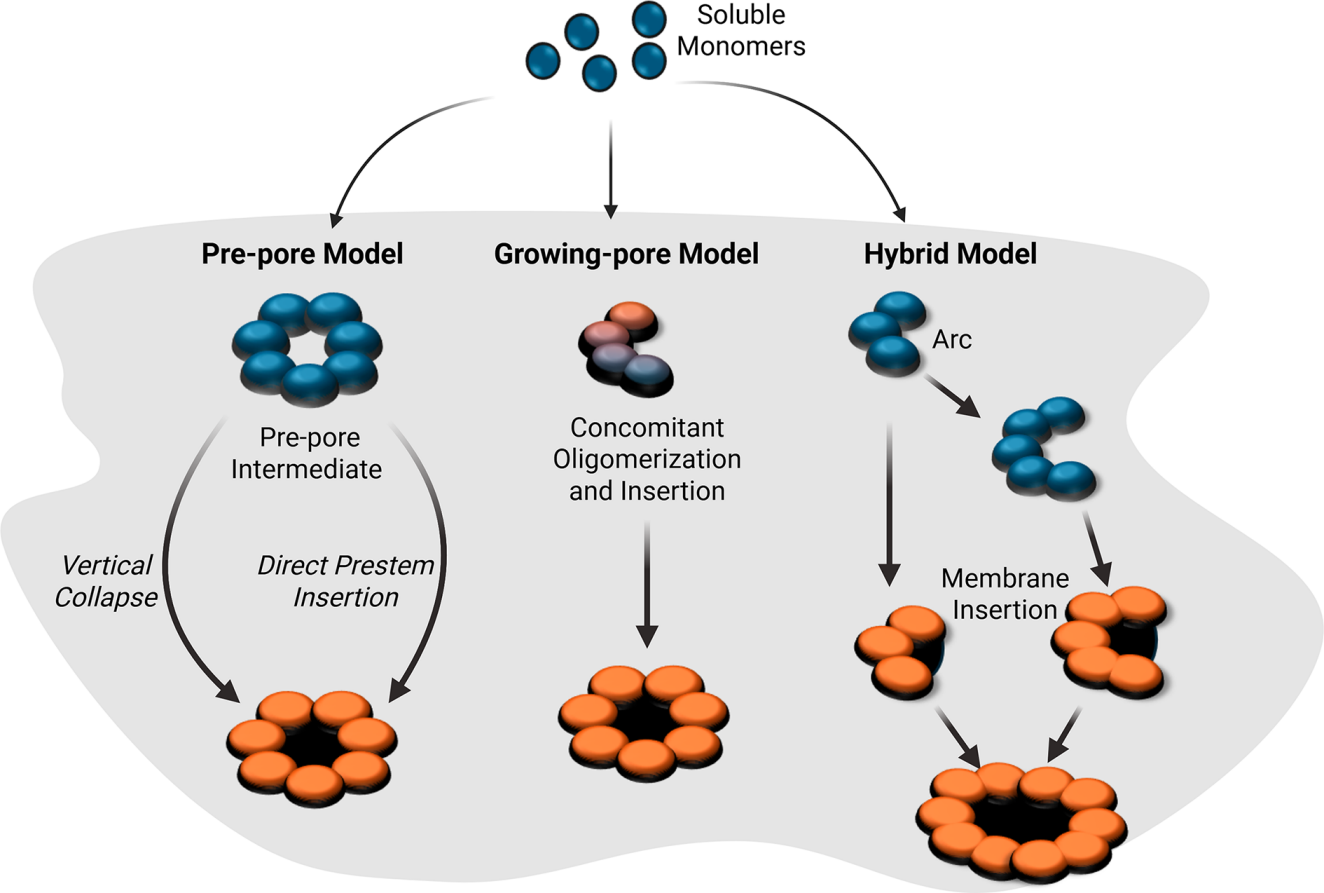
Mechanistic models of pore-formation by PFTs Most PFTs are secreted as soluble toxin monomers that bind to the target membrane and form transmembrane pores through three principal pathways. In the pre-pore model (left panel), membrane-bound monomers first assemble into a non-inserted oligomeric pre-pore intermediate, followed by a concerted conformational change either through vertical collapse or direct prestem insertion to form the transmembrane pore. In the growing-pore model (middle panel), oligomerization and membrane insertion occur concomitantly, with protomers inserting sequentially without a metastable pre-pore intermediate. In the hybrid model (right panel), partial oligomers or arc-shaped intermediates insert into the membrane and may subsequently recruit additional protomers or fuse with other arcs to complete the pore. Orange structures represent membrane-inserted pore states, whereas blue structures indicate soluble or membrane-bound non-inserted intermediates.

The general mechanism by which PFTs integrate into the lipid bilayer to form transmembrane pores follows a stepwise process that includes membrane binding, oligomerization, and membrane-insertion. The intricate molecular details of these three events vary across PFT families, depending on the structure and function of the toxin. PFTs are secreted by the bacteria as water-soluble monomers. The initial step involves binding non-covalently with the membrane receptors and anchoring to the surface. This membrane association concentrates the dispersed monomers at the binding site to facilitate oligomerization, which is followed by the final step of membrane insertion [4,9]. Structural studies highlight that oligomerization of PFT monomers can be governed by different mechanisms other than lateral diffusion in the membrane plane. For example, in the case of *Staphylococcus aureus* α-hemolysin (Hla), binding to its receptor nucleates the oligomerization by stabilizing the protomer interface required for heptamer formation [10,11]. However, some PFTs, like cytolysin A (ClyA), undergo major structural rearrangements that drive oligomerization [12]. Moreover, oligomerization can also be promoted by allosteric regulators such as lipids or cooperative assembly kinetics [13–15]. The intricate molecular details of pore formation vary across PFT families, depending on the structure and function of the toxin. Pore formation by PFTs generally follows three mechanistic pathways: (i) a pre-pore model, where oligomerization results in the formation of a transient structure, generally termed as ‘pre-pore’; (ii) a growing-pore model, where oligomerization and membrane insertion occur simultaneously; and (iii) a hybrid model, in which incomplete oligomeric rings or arcs are sufficient to destabilize and lyse the membrane (Figure 1) [9,16,17]. In the first model, pre-pore assembly of many β-PFTs undergoes a vertical collapse to achieve membrane insertion. In the case of CDCs, soluble monomers bind to cholesterol-rich membranes, oligomerize, and undergo a cooperative vertical collapse that leads to the insertion of β-hairpins into a transmembrane β-barrel [13,18]. However, pre-pore formation in other β-PFTs, such as the aerolysin-like toxins, Hla and *Vibrio cholerae* cytolysin (VCC), can proceed without a large vertical collapse, instead involving prestem rotation, local refolding, and direct insertion into the membrane [19–21]. Many other PFTs, especially α-PFTs, follow a ‘growing-pore’ model in which oligomerization and membrane insertion occur without a stable pre-pore intermediate. For example, membrane-binding-dependent conformational changes in ClyA family toxins lead to exposure and extension of previously buried α-helices, which insert into the target lipid bilayer [12,22]. Here, membrane insertion proceeds sequentially rather than by cooperative collapse. Some PFTs (majorly CDCs) also form incomplete ring structures like arcs and slits, which may be lytic even without full-ring closure, or they may fuse together to form the closed ring assemblies [13]. This ‘hybrid model’ indicates that oligomerization and membrane insertion can occur progressively, combining features of both the growing-pore and pre-pore mechanisms. The process of pore formation is typically rapid and tightly regulated and often dictates the outcome of infection. In the following sections, we trace the dynamic journey of PFTs from their initial engagements with the target membrane and follow the possible pathways thereafter.

## The first encounter: surface interactions and host receptors

**Figure 2:**
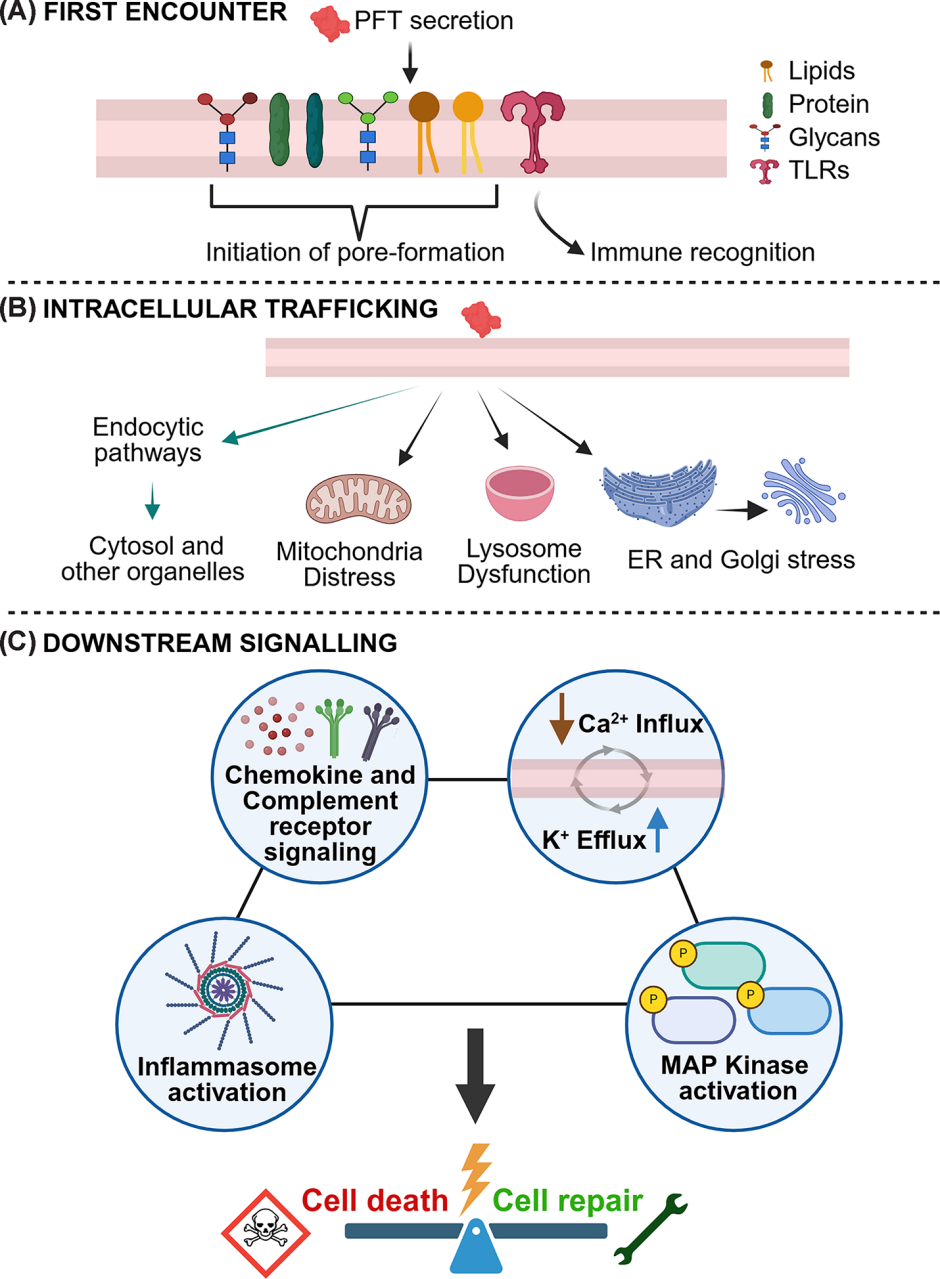
Mechanisms of PFT interaction with host cells and downstream signaling pathways (**A**) The first encounter—PFTs, after secretion by the pathogens, mediate their initial host cell recognition through direct binding to membrane components such as lipids, proteins, and glycans, which initiates the process of pore formation. Additionally, in the case of immune cells, certain TLRs can recognize PFTs as PAMPs and thereby initiate the downstream innate signaling. (**B**) Intracellular trafficking and cellular stress—Internalization of PFTs by host cells can occur via various endocytic pathways as well as retrograde trafficking pathways. Release of PFTs into the cytosol through specific endocytic pathways can disrupt cellular homeostasis and indirectly or directly affect the integrity of organelles. Direct localization of PFTs to different organelles can trigger membrane disruption and affect overall structural and functional integrity. For example, mitochondrial damage can lead to impaired energy production and release of pro-apoptotic factors. Impairment of lysosomal function affects proteolytic processes and cellular waste disposal. Further, distress of the ER and Golgi hampers protein folding and processing. (**C**) Downstream signaling and cellular outcomes—PFT-mediated interaction and pore formation can trigger a cascade of host cell signaling events that are crucial for determining the cellular responses. Pore formation results in the ionic imbalance, typically the influx of Ca^2+^ and efflux of K^+^, both of which are critical mediators of various cellular responses involved in cell death and cell repair pathways. Other major signaling pathways, include chemokine and complement receptor-based immune responses, MAP kinase activation, and activation of various inflammasomes. The concerted action of these upstream interactions, intracellular events, and downstream signaling pathways ultimately leads to either cell death (e.g., via apoptosis, necrosis, or pyroptosis) or cell repair mechanisms, which attempt to restore cellular integrity and function following PFT damage. The balance between these outcomes dictates the pathogenesis and resolution of PFT-mediated infections. Image Created in BioRender (https://BioRender.com/5qx9epl).

Once a PFT is secreted, it begins its journey with specific and opportunistic binding to host cell surfaces or organelle membranes [23]. PFTs can employ any of the cell-surface entities, such as lipids, proteins, and glycans, to bind and initiate the process of pore formation (Figure 2A) [16,24]. Lipids are the crucial partners for PFTs because they serve both as direct receptors and as membrane organizers that facilitate toxin binding and insertion [25,26]. There is increasing evidence that target membranes and host receptors actively regulate toxin activation and oligomerization and allosterically trigger conformational switches that promote efficient pore formation. CDCs bind to membrane cholesterol through conserved motifs, often in conjunction with glycan recognition [27,28]. Some toxins, such as ClyA and lysenin (a non-bacterial PFT found in earthworm *Eisenia fetida*), target cholesterol- and sphingomyelin-rich microdomains for efficient binding and pore formation [29,30]. Proteinaceous receptors play a significant role in providing the specific binding. PFTs like Hla, *Clostridium perfringens* β-toxin, and RTX family members directly bind to host proteins such as ADAM10, CD31, CD11/CD18, or Nectin-2 [15]. Binding of Hla with ADAM10 (a disintegrin and metalloproteinase 10) is an excellent example of receptor-mediated pore maturation. ADAM10 stabilizes the monomer and spatially organizes monomers for heptamer formation, hence promoting oligomerization into a pre-pore ring. ADAM10-mediated pore formation of Hla dictates the cellular tropism and lytic potency [10,31,32]. Similarly, structural and functional studies of leukocidin family toxins have revealed sequential binding to immune receptors such as CD11/CD18, leading to the formation of hetero-oligomers and selective targeting of leukocytes [33]. Receptor-mediated activation can also be observed in aerolysin-like toxins, where interactions with GPI-anchored receptors trigger conformational transitions required for oligomerization and membrane insertion [19,34]. Some bacterial PFTs, including VCC, *Vibrio vulnificus* hemolysin (VVH), and certain members of the CDC family, interact with glycan structures on host membranes, such as GPI-anchored proteins or specific sugar moieties such as glycerol, N-acetyl-d-galactosamine, and N-acetyl-d-lactosamine [15]. These glycan interactions not only facilitate membrane binding but also contribute to the cellular tropism of the PFTs [15,27]. The multifaceted receptor interactions of PFTs expand the range of host cells susceptible to these toxins and enhance their cytotoxic potency during infection. Host receptors play structural roles beyond simple docking. For example, cholesterol supports pore formation in VCC and ClyA by stabilizing oligomeric assemblies, in addition to membrane association [30,35]. Moreover, cholesterol is also shown to be essential for membrane insertion in CDCs such as PLY, ILY, and SLO [36,37]. However, in the case of LLO (a member of the CDC family), cholesterol is essential for the step of oligomerization, not membrane insertion [26]. These differences highlight that detailed structural and biochemical studies of toxin-membrane interactions are needed to understand the molecular mechanisms of pore formation. Studies on PFT–receptor complexes provide mechanistic insight into how receptor identity dictates oligomer stoichiometry, cellular tropism, immune signaling, and even sub-lytic outcomes. Such findings establish receptor engagement as a key regulatory checkpoint in PFT molecular function.

Beyond these conventional cell surface receptors, immune cells can recognize PFTs as pathogen-associated molecular patterns (PAMPs) through the pattern recognition receptors (PRRs) present on their surfaces to initiate the host-defense responses [5]. Toll-like receptors (TLRs) are the most common PRRs that recognize PFTs. Recognition of pneumolysin (PLY) by the TLRs has been shown to regulate inflammatory responses during infection of *Streptococcus pneumoniae* [38]. While studying how PFTs are recognized by TLRs, a novel TLR1/4 heterodimer has been discovered recently. VCC was shown to employ this novel TLR heterodimer to induce pro-inflammatory responses in dendritic cells. Interestingly, for the VCC-mediated pro-inflammatory signals, macrophages also employ additional receptors, such as TLR2/6, along with TLR1/4 [39,40]. TLR4 plays a significant role in the recognition of various CDCs such as PLY, anthrolysin O (ALO), and listeriolysin O (LLO) [38,41]. Overall, these findings highlight the diverse and dynamic nature of TLR-mediated recognition of PFTs and reveal both established and novel receptor pairings that fine-tune the host immune response to different bacterial pathogens. In addition to the membrane association, PFTs can also get internalized in the cells, which can be a crucial determinant for the cellular fate. Hence, it is important to understand what happens once/if a toxin enters the cell and how it is trafficked and whether it targets intracellular compartments to modulate host processes.

## Intracellular fate: trafficking and translocation into organelles

PFTs traditionally have been known for disrupting the plasma membrane, while recent works in this field have shown that many PFTs reach the host cell cytosol to fully exert their pathogenic effects, including modulation of intracellular signaling and induction of cell death [42]. Beyond their initial membrane interactions, numerous PFTs follow complex intracellular trafficking routes and selectively target specific organelles, including mitochondria, lysosomes, Golgi apparatus, and endoplasmic reticulum (ER), expanding their roles in bacterial pathogenesis (Figure 2B). In some instances, toxin binding to the host cell surface enables direct translocation across the plasma membrane into the cytosol [43]. Certain PFTs can be internalized via endocytosis and enter endosomal compartments, and then can escape into the cytosol by perforating the endosomal membrane [44]. Other PFTs can also take advantage of retrograde trafficking pathways, moving from endosomes to organelles like the Golgi apparatus and ER, and, in some cases, even reaching the mitochondria or the nucleus (rarely) [42]. Their intracellular journey is dependent on several factors, including the toxin’s structure, post-translational modifications, mechanism of interaction with the host receptors, and the accessibility of the host’s trafficking machinery [45–47]. This flexibility in how bacterial proteins/toxins translocate and target different organelles highlights the clever ways by which pathogens can manipulate host cell functions to their favor. To get inside the host cells, bacterial PFTs use a range of endocytic pathways, such as clathrin- or caveolin-mediated uptake, macropinocytosis, lipid raft-dependent entry, and dynamin-driven routes. The exact path it chooses often depends on the type of toxin and the specific conditions within the host cell [2,48,49]. Table 1 summarizes the bacterial PFTs that undergo endocytosis and follow vesicular trafficking inside the host cells, including several well-studied examples from various bacterial species.

**Table 1:** Endocytic pathways involved in the internalization of PFTs

Endocytic pathway	Example of PFTs	Host cell types	References
Clathrin-mediated	α-toxin, LLO, Anthrax PA	Epithelial, endothelial, various non-phagocytic cells	[50–52]
Caveolin-mediated	MakA, LLO	Endothelial, epithelial, and muscle cells	[53,54]
Macropinocytosis	α-toxin	Non-phagocytic cells (epithelial/endothelial)	[31,55]
Lipid raft-mediated	VacA, Anthrax PA	Gastric epithelial, immune, endothelial	[52,56]
Dynamin-mediated	α-toxin	Ovarian cells	[57]

Some PFTs also take retrograde routes to intracellular organelles such as the ER and Golgi apparatus or localize to organelles like mitochondria, nucleus, and lysosomes, where they disrupt organelle function and enhance bacterial pathogenicity. To access their intracellular targets, certain bacterial toxins exploit the host cell’s retrograde trafficking pathway. The ionic imbalance resulting from membrane perforations can induce ER stress and activate the unfolded protein response (UPR). Furthermore, persistent stress can lead to ER structural alterations, including vacuolation and fragmentation, and can ultimately lead to apoptosis if homeostasis is not reestablished. Both trafficking and membrane-disrupting toxins often affect the ER, either by passing through it or causing stress there, linking their actions to the cell’s overall stress and death responses. PFTs can also translocate to lysosomes to compromise the structural integrity of the membrane. This can lead to the release of lysosomal enzymes such as cathepsins into the cytosol that can ultimately disrupt cellular homeostasis and cause cell damage. This strategy helps PFTs bypass extracellular defenses and exploit intracellular compartments to enhance bacterial survival and virulence. LLO from *Listeria monocytogenes* and MakA from *V. cholerae* mediate lysosomal membrane permeabilization after getting endocytosed, facilitating bacterial escape from the phagosome into the host cytosol [53,58]. TDH from *V. parahaemolyticus* can also translocate to the lysosome, cause lysosomal membrane permeabilization (LMP) in the host cells, and even play an important role in TDH trafficking to the host cell mitochondria [59]. Mitochondria are central regulators of programmed cell death, and thus they are one of the prominent targets of bacteria to enhance their pathogenesis. Hence, several bacterial PFTs localize and disrupt its structure and function. For example, *Helicobacter pylori* VacA induces mitochondrial fission, loss of membrane potential, ATP depletion, and recruits pro-apoptotic proteins like Bax, Bak, and cytochrome c, leading to apoptosis [60]. *Listeria monocytogenes* LLO disrupts mitochondrial dynamics and increases host susceptibility to infection [61]. β-PFTs such as PLY and PVL also cause mitochondrial damage and apoptosis [62,63]. *Vibrio cholerae* VCC impairs mitochondrial function and can trigger cell death independent of its pore-forming activity [64]. Similarly, TDH induces caspase-independent death via mitochondrial dysfunction and activation of ROS, Ca^2+^, apoptosis-inducing factor (AIF), poly (ADP-ribose) polymerase (PARP-1), and endonuclease G [59]. Altogether, it is evident that PFTs do not just target the plasma membranes; they can continue to act after internalization. Further, beyond structural engagement and internalization, the interaction of PFTs with membrane can serve as a trigger for host cell signaling, either through direct interaction with a receptor or as a consequence of pore formation. Hence, the encounter with PFTs can set off a cascade of molecular alarms, which are discussed in the following sections.

## Balancing defense and evasion: immune signaling and cell death pathways

Once PFTs interact and associate with target cells, several signaling pathways can be triggered (Figure 2C). At first, PFT-induced membrane perforations allow the selective passage of specific ions such as calcium and potassium across the membrane. Calcium influx through the pores can activate multiple cell death pathways, neutrophil lysis, reactive oxygen species (ROS) production, proinflammatory cytokine release, and granulocyte chemotaxis [65–68]. This pore formation-induced Ca^2+^ influx even activates cytoskeleton-dependent repair mechanisms. For example, myeloid cells respond by using Ca^2+^-dependent signaling to shed damaged membrane regions as microvesicles, which aids cell survival and limits the immune detection, which ultimately favors the bacterial persistence [69,70]. On the other hand, the loss of cytosolic potassium simultaneously disrupts metabolic homeostasis, which activates the innate immune response, often culminating in proinflammatory cell death [6,71,72]. These ion fluxes act as the first alarm signals that activate a network of host signaling pathways to counter infection.

Being a critical virulence factor of various pathogens, PFTs attempt to enable the prolonged survival and persistence of the pathogen within the host, without alarming the immune system. PFTs can modulate the production of cytokines to create an immunosuppressive environment [2]. For instance, after invading macrophages, p38 MAPK pathway is activated by the β-hemolysin/cytolysin (βh/c) toxin secreted by group B *Streptococcus*, leading to an increased production of anti-inflammatory cytokine IL-10 and reduced secretion of pro-inflammatory cytokine IL-12 and nitric oxide synthase-2 (NOS2) [73]. Similarly, *S. aureus* α-toxin weakens the type 1 immune response by modulating CD4+ T cell polarization toward Th17 over Th1 [74]. *Escherichia coli* α-hemolysin inhibits IL-1β secretion, further suppressing inflammation and bacterial clearance [75]. In *Caenorhabditis elegans*, inhibition of p38 was shown to increase susceptibility to aerolysin-induced mortality, which highlights its protective role [76]. In contrast, certain pathogens exploit this same pathway for bacterial adherence and immune pathway modulation, as observed in the manipulation of MAPK phosphorylation by *L. monocytogenes* LLO [77]. PLY exposed to epithelial cells exploits p38 activity to secrete IL-8, which is a potent neutrophil attractant, highlighting the significance of MAPK pathway activation in the host defense mechanism [78].

PFTs can also specifically target immune cells by exhibiting tropism for chemokine and complement receptors. *Staphylococcal* γ-hemolysin (HlgAB and HlgCB) targets chemokine and complement receptors (CXCR1, CXCR2, CCR2, C5aR, and C5L2) on phagocytic cells, contributing to the severity of bacteremia [79], while leukotoxin LukED binds CCR5 on myeloid and T cells and drives the depletion of immune cells [80]. Depending on the local receptor expression and cellular composition of the infected tissue, PFTs can have tissue-specific effects on immune responses. *S. aureus* α-toxin disrupts epithelial barriers through interaction with ADAM10 in skin infections, but it also dampens IL-1β in lungs, thus offering protection against severe pneumonia [81]. *S. pneumoniae* PLY also exhibits dual functions: it triggers inflammation in the upper respiratory tract to facilitate spread, while variants with impaired pore formation avoid triggering strong immune responses in the lungs to favor the bacterial persistence [82]. Additionally, PFTs exploit host-derived microvesicles for immune modulation. Macrophages internalizing PLY-carrying vesicles polarize into CD14^+^MHCII^low^CD86^low^ phenotypes, which amplify Gram-positive responses while diminishing Gram-negative ones, potentially aiding persistence [83]. Inflammasomes are multiprotein complexes that play a crucial role in inflammation and cell death and restrict the infection by containing pathogens, thereby maintaining tissue integrity [84]. During intracellular infections, PFTs facilitate inflammasome activation upon bacterial escape into the cytosol, as observed with *Listeria monocytogenes* infection, where LLO triggers NLRP3 and AIM2 pathway activation [85,86]. Disruption of these inflammasomes is found to be associated with increased bacterial colonization and mortality in the host during *S. pneumoniae* infection, illustrating the crucial protective role of inflammasomes in the infection scenario. Thus, targeting inflammasome activation could lower inflammation levels and improve survival and prognosis for patients, presenting a promising therapeutic approach for some infections [87].

Cells activate several signaling pathways in response to PFT attack, and sometimes the cells may not be able to resist the toxin attack, and finally they undergo programmed cell death along with osmotic lysis. VCC induces programmed cell death in intestinal epithelial cells [88], and, notably, even the mutants of VCC lacking pore-forming ability can activate the apoptotic pathway [64]. Additionally, thermostable direct hemolysin (TDH) from *V. parahaemolyticus* can induce non-classical, caspase-independent programmed cell death [59]. ε-toxin from *C. perfringens* activates apoptosis in lung cells via a caspase-3-dependent mechanism, highlighting the potential for aerosolized toxins to initiate apoptotic pathways, which poses a major threat in the context of bioterrorism [89]. Additionally, PFTs can also trigger pyroptosis, necroptosis, and ferroptosis, each contributing to distinct cellular responses and are important in contexts of inflammation and infection. For instance, several PFTs such as *Vibrio proteolyticus* hemolysin (VPRH), *Clostridium septicum* α-toxin, and *E. coli* hemolysin are known to activate the pyroptotic pathway [5,90–92]. Necroptosis is activated by PFTs, particularly in epithelial and immune cells [71,93]. PLY from *S. pneumoniae* induce necroptosis in respiratory epithelial cells, which contributes to tissue injury during infection [94]. These regulated cell death pathways trigger various signaling pathways and serve as an alarm for the neighboring cells.

The host has also evolved several strategies to comprehend PFT-induced membrane damage. For example, the target cells shed damaged segments of the membrane of infected macrophages containing toxins as microvesicles [83,95]. Toxin endocytosis, a lesser-understood process, is also employed by cells to internalize PFTs via endocytosis, which leads to minimization of surface-bound toxins [95]. Additionally, cellular integrity is achieved through fusion of internal vesicles with the membrane to seal pores [96]. Besides all these signaling, there are several studies highlighting that pore formation itself is not always essential for their downstream effects.

## Pore-independent activities of PFTs

Emerging research shows that PFTs can influence host cells in ways that do not even require pore-forming activity. There are significant reports suggesting that mutants that cannot form pores can still bind to the cells and trigger signaling. For example, mutants of VCC that lack pore-forming activity can induce caspase-dependent apoptosis as well as mitochondrial membrane damage. Two mutants were employed in the present study, one lacking the ability to oligomerize and the other deficient in membrane insertion but capable of forming abortive, non-hemolytic oligomers. Interestingly, both of them displayed a prominent propensity to translocate to the target cell mitochondria and exhibited cytotoxicity comparable to wild-type VCC, demonstrating that pore formation is not essential for VCC-mediated cytotoxicity in epithelial cells [64]. Similarly, two of the helix 4 mutants of Cry1Aa from *B. thuringiensis* were also reported to display cellular responses leading to cytotoxicity, despite the loss in their pore-forming ability [97]. In another study, revertant mutants were generated to get important insights into pore formation mechanism of Cyt2Aa1 from *B. thuringiensis*. These mutants displayed larvicidal activity comparable to the wild type despite significantly less hemolytic activity [98]. Another compelling example of pore-independent functions of a PFT is PLY. There is a naturally occurring non-hemolytic variant, PLY-NH, where the pore-formation is inhibited because of specific mutations Y150H and T172I. Despite this loss in pore-forming activity, PLY-NH could interact with host cells and significantly modulate immune responses, suggesting that certain immunomodulatory functions of PLY can occur independent of pore formation [82]. However, it is important to be cautious while exploring and exploiting such pore-independent functions of PFTs. Sometimes, the residual activity, time dependency, or difference in the sensitivity of different assays can mislead the observations.

PFTs are also being developed as toxoid-based vaccines and are being explored as adjuvants due to their ability to stimulate the immune system. For example, a full-length LLO toxoid (LLO^T^) was generated with mutations in the cholesterol recognition motif, and utilizing the adjuvant cholera toxin, an attempt was made to develop a new experimental vaccine. This LLO^T^ retained all antigenic epitopes, but LLO toxicity was eliminated [99]. Similarly, sticholysin (St) II of *Stichodactyla helianthus* was encapsulated with ovalbumin and transformed into liposomes to check the effectiveness of cytotoxic T lymphocytes. It was found that these St protein-treated mice contained greater memory cells [100]. In such exploration of vaccine and adjuvant development, non-hemolytic variants of PFTs with immunogenic ability are being regularly employed, suggesting the functional diversity of PFTs, independent of pore-formation.

## From structure to function: how technology is revolutionizing the PFT studies

The comprehensive understanding of the structural features of PFTs as well as insights into the intricate molecular details of the mechanism of pore formation are essential for understanding disease progression and designing effective therapies. Traditional biochemical assays along with X-ray crystallography and cryo-electron microscopy (cryo-EM) have majorly advanced the structural and functional characterization of PFTs. Technological limitations often restricted these methods from capturing static structural snapshots. The kinetics, interactions with the membrane, and the mechanism of pore formation remained underexplored for many PFTs. Fortunately, advances in cryo-EM, AlphaFold, lipidomics, and single-molecule techniques have transformed how we understand the biology of PFTs. We can now visualize these proteins in action and dissect their interactions at exceptional resolution. In this section, we briefly discuss how technological advancements have enriched our understanding of PFT biology.

### Cryo-electron microscopy and atomic force microscopy

Historically, cryo-electron microscopy has generated low-resolution static structures of oligomeric transmembrane complexes. However, advancements in cryo-EM have revolutionized the study of PFTs to a whole new level [101]. These advancements primarily involve sample preparation, data collection, and computational algorithms; consequently, they yield several new structures that offer atomic-level insights into the molecular mechanisms of pore generation [24,101]. A quick view of entries of PFT structures in the Electron Microscopy Data Bank (EMDB) reveals a total of over 50 entries, of which more than 40 have been reported in the last five years, and around 15 structures have been deposited in just the past year. These numbers underscore how cryo-EM has revolutionized our understanding of the mechanism of pore formation and significantly filled the knowledge gaps in mechanistic insights.

Earlier, conventional atomic force microscopy (AFM) imaging mainly gave information about how the PFTs are inserted and oligomerized in the membrane by providing their topographic maps. The conventional AFM records static images or extremely slow reorganization processes of PFTs as they collect images in a minute range. To overcome this limitation, high-speed AFM has been used, which provides high spatio-temporal resolution within a sub-second range [102]. Thus, it provides information about the transitioning of PFTs from pre-pore to pore state, their oligomeric assemblies, and kinetics [103,104]. Further, AFM studies also provide information about the lipid composition-dependent membrane organization. This provides major insights into how the organization of membranes facilitates the pore formation and how PFTs can affect the membrane organization during the course of pore formation. Therefore, the mechanistic insights provided by AFM are not limited to the protein alone but also take the membrane into account, offering a comprehensive view of the entire process [102].

### Artificial intelligence and molecular dynamics simulations

Deep learning tools like AlphaFold and RoseTTAFold are employed for the prediction of protein structures, including that of PFTs. A recent study utilized AlphaFold-guided mutagenesis to explore the residues in TspA, which are crucial for the T7SS-dependent pore formation [105]. AI is also assisting in mining genomic databases to identify novel PFT families [106]. Furthermore, molecular dynamics (MD) simulations have long guided molecular-level understanding of PFTs, primarily the assembly and the pre-pore-to-pore rearrangement [107,108]. However, the wide extracellular region of assemblies and the substantial number of atoms in an oligomeric pore provide some difficulties for MD simulations and make computational modeling challenging [108]. To overcome these challenges, atomistic and coarse-grained simulations are often used for studying the interaction between protein and lipid, dynamics in lipid bilayers, and larger assemblies of oligomers. For example, MD simulations have revealed the crucial interaction of CDCs such as PLY and LLO with membranes and their specificity toward cholesterol [108–111]. Thus, simulations offer a potent tool for analyzing atomistic characteristics, quaternary interactions, and intricate aspects of PFTs.

### Single-molecule imaging and microfluidics

Single-molecule imaging has been employed to examine the oligomerization of proteins with molecular-level resolution. It provides spatio-temporal information at the nanoscale. Single-molecule FRET (smFRET) is one of the single-molecule-based approaches that measures the inter- and intramolecular distances in real time with great precision [112]. For example, structural changes in LLO in the presence of GUVs and also dynamics in membranes during pore formation have been revealed utilizing smFRET [110,113]. Another area of growing importance is microfluidics, which has the advantage of manipulating tiny volumes on microscale chips. It acts like a mini-lab-on-chip, which is employed in the synthesis of liposomes, protein purification, bacterial transformation, and protein isolation. Additionally, it helps with cryo-EM sample preparation by controlling sample flow, blotting, and vitrification on chip [114–116].

### Lipidomics, glycomics, and synthetic membrane systems

Development of lipidomic and glycomic platforms has assisted significantly in our understanding of PFT specificity. A recent study employed glycan arrays to reveal that all major CDCs utilize glycans as cellular receptors, providing novel insights into the cellular tropism of CDCs [27]. Artificial membrane systems such as liposomes and nanodiscs are widely used to study PFTs in their mimicked native membrane environment. The use of liposomes as biomimetic membranes has proven instrumental in characterizing structural features, binding interactions, and pore-formation processes of PFTs [21,24,117]. As an alternative model system, nanodiscs offer a few advantages over detergents and liposomes to examine membrane proteins, including a more native-like lipid environment, improved stability, and defined size and composition. The nanodiscs enable a natural lipid environment for membrane proteins, facilitating structural analysis using methods such as cryo-EM [113,118]. In the case of PFTs, a major advantage offered by nanodiscs is the reduction of conformational heterogeneity, which is a significant technical limitation in cryo-EM studies of many pore complexes.

## Advances in the discovery of novel pore-forming toxins

Recent advances highlight that nature harbors a repertoire of PFTs with diverse structural architectures and unique mechanisms that are yet to be explored. Using cutting-edge technologies, researchers have identified new toxins and candidate toxin genes within the microbial communities, though many are yet to be structurally and functionally defined. A prominent discovery is the family of CDC-like proteins (CDCL), which are found in gut microbes such as *Bacteroides spp.* [119]. They are structurally similar to CDC families, but unlike CDCs, CDCLs are secreted as a tool to win the competition by killing other bacteria residing in the same environment. These toxins form giant antibacterial pores (150–200 Å) and function as a two-component system that involves proteolytic activation of its two components (CDCL-L and CDCL-S). The structure of the CDCL monomer and pore complex was recently published, providing mechanistic insights about its mode of action [119]. There are other bacterial PFTs that have been recently discovered and characterized as a part of a bacterial competition system. These toxins get delivered majorly via bacterial secretion systems like T6SS and T7SS. Such antibacterial PFTs include Tme1/Tme2 (*V. parahaemolyticus*), Ssp6 (*Serratia marcescens*), TspA (*S. aureus*), and Tse4 (*Pseudomonas aeruginosa*), which were recently characterized for their roles in microbial competition [15,120]. These recent discoveries underscore a trend in identifying PFTs employed by bacteria (other than colicins) to compete with each other. Furthermore, pore-formation mechanisms of tripartite (belonging to the group of α-PFTs) were recently characterized for multi-component systems, including MakABE from *V. cholerae*, AhlABC from *Aeromonas hydrophila*, and SmhABC from *Serratia marcescens* [121–124]. These PFTs target host cell membranes via GPI-anchored proteins or lysosomal V-ATPase and require all three components for maximal cell lysis. Distinct structural features of these PFTs challenge the existing models of pore symmetry and the membrane interaction mechanism. Overall, these discoveries highlight the growing recognition of PFT diversity, particularly in bacterial antagonism and host–pathogen interactions.

## Emerging therapeutic strategies targeting PFTs

A wide range of pharmacological strategies have been developed to target the pore-forming functionality of bacterial PFTs, with the purpose of neutralizing their cytolytic effects associated with the pathological conditions [125,126]. Among these, small-molecule inhibitors, neutralizing antibodies, and polyvalent inhibitors have also been explored as promising therapeutic strategies to tackle PFT-mediated pathological conditions [127–136]. Additionally, synthetic receptor-like decoys have also been employed to mimic the natural host receptors and bind to PFTs, preventing the toxins from interacting with target cells. This strategy has been particularly effective against CDCs like SLO, LLO, and PLY [137–139]. Beyond direct toxin neutralization, both small molecules and monoclonal antibodies have been employed to block the receptor-mediated uptake of toxins, such as *S. aureus* LukED and certain CDC members, by the target cells [80,140,141]. Furthermore, several PFTs (e.g., CDCs and Hla) have been explored as vaccine candidates. The nontoxic variants of these toxins, while still maintaining immunogenicity, can effectively prime the host immune system and provide protective immunity against bacterial infections [142,143]. Table 2 provides a comprehensive summary of various bacterial PFTs along with the therapeutic strategies employed to inhibit their activity, ranging from direct neutralization to receptor blockade and vaccine development.

**Table 2:** Therapeutic approaches employed for PFT neutralization

PFTs	Approaches to PFT neutralization	References
Hla (*S. aureus*) PLY (*S. pneumoniae*) SLO (*S. pyogenes*) LLO (*L. monocytogenes*)	- Small molecules or peptides targeting PFTs or inhibiting PFT oligomerization - Decoy-based neutralization and nanosponge systems	[127–129,137–139,144]
Hla, LukSF-PV, LukED, HlgAB, HlgCB, LukGH (*S. aureus*)	Monoclonal antibody-mediated neutralization of PFT activity	[131–135]
LukED (*S. aureus*) ILY (*S. intermedius*)	Agents inhibiting host cell receptor or uptake of PFTs	[80,140,141]
Hla (*S. aureus*) PA (*B. anthracis*) C2 (*C. botulinum*) iota toxin (*C. perfringens*)	Polyvalent inhibitors obstructing the PFT-mediated pore-formation	[136,145]
SLO (*S. pyogenes*) Hla (*S. aureus*)	PFT (Toxoid) vaccine candidates	[142,143]

PFTs have demonstrated exceptional potential as therapeutic agents, especially in the treatment of cancer [146]. PFTs possess a multitude of unique biochemical attributes that can be exploited for the therapeutic purposes in treating cancerous cells [146–148]. These include the ability of PFTs to form pores in various membranous compartments (including plasma membrane and intracellular cell organelles). Thus, multiple binding preferences of PFTs identify them as potential candidates for manipulating cancer cell function. Additionally, the structural flexibility and bioengineering potential of PFTs help in the development of customizable therapeutic agents in targeting tumor cells while minimizing non-specific activity. Furthermore, the inherent ability of PFTs to lyse the cells has demonstrated remarkable potential as therapeutic agents for targeting cancer cells [147–149]. Interestingly, early studies indicated the use of bacterial systems for delivering PFTs directly to the tumor-affected regions. This has been particularly reported in the case of *S. aureus* Hla, which was cloned into *E. coli* and subsequently injected into murine tumor tissues. The engineered *E. coli* successfully secreted Hla within tumor microenvironment. The potent cytolytic activity of Hla in mouse models was demonstrated by immunohistochemical analysis, which showed that Hla caused widespread necrosis in the tumor tissues [149]. Additionally, other PFTs have also demonstrated a natural tendency to bind specifically to cancer-associated cell surface markers. In particular, *C. perfringens* enterotoxin (CPE) has been found to interact specifically with the tight junction proteins such as claudin-4 and claudin-6, which are frequently overexpressed in certain human cancers [146,150,151].

More recently, protein engineering has immensely contributed toward the modification of PFTs to improve specificity for targeting cancer cells. One approach involves fusing target peptides to the toxin and directing its cytotoxic effects towards cells that overexpress specific receptors. A recent report suggested the fusion of the luteinizing hormone-releasing hormone (LHRH) receptor ligand to LLO and *Lysinibacillus sphaericus* BinB. This approach has been used to selectively target LHRH receptor-positive cancers, such as ovarian and breast cancers [147,148]. Another innovative strategy involves engineering a fusion construct of *S. aureus* Hla to target tumors that overexpress matrix metalloproteinase-2 (MMP-2) [152]. In this context, galectin-1 was fused to Hla to enhance its binding specificity toward cancer cells overexpressing glycoconjugates [152]. Additionally, to enhance treatment precision, specially engineered mutants of Hla were designed such that they exhibit potent cytolytic activity only in the tumor microenvironment, upon cleavage by MMP-2 (overexpressed in tumors), and remain inactive under normal conditions [152,153]. A similar strategy was employed in another study for generating engineered immunotoxins that target furin-overexpressing cancer cells [154]. Furin is a proprotein convertase and often up-regulated in various tumors. Selective cytotoxicity in cancer cells was achieved by engineering an actinoporin produced by the sea anemone *Actinia fragacea*, Fragaceatoxin C (FraC), with tumor-targeting molecules and a soluble domain that blocks pore-forming activity, keeping it inactive until furin cleaves it [154,155].

**Figure 3:**
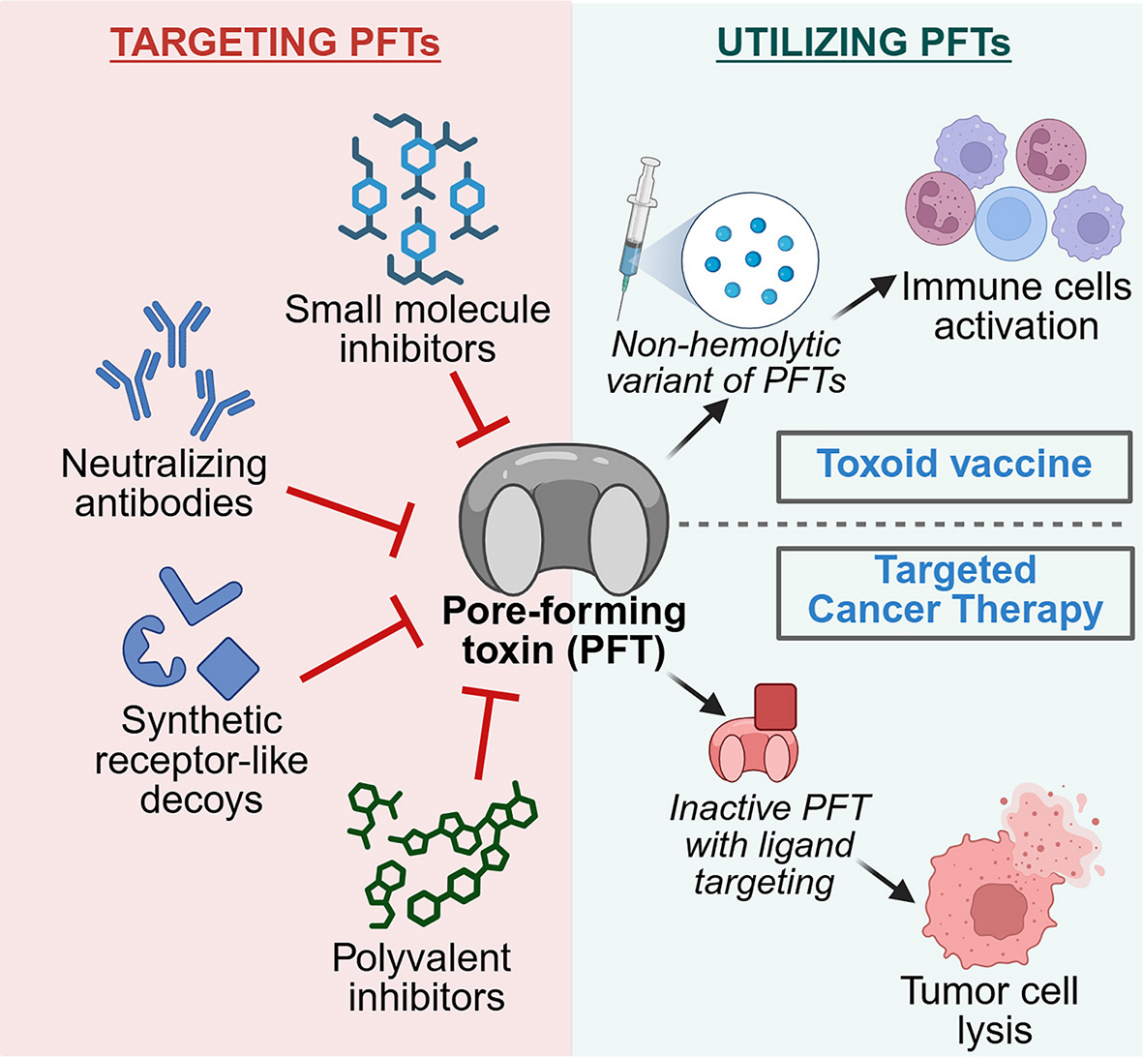
Overview of therapeutic strategies developed to combat PFT-mediated complications and harnessing PFTs for vaccine design and targeted cancer therapy The left panel illustrates the use of numerous synthetic molecules for mitigating PFT-induced pathological conditions. Small molecule inhibitors prevent oligomerization and pore formation, monoclonal antibodies block receptor binding or neutralize PFT activity, receptor antagonists prevent toxin docking, and polyvalent scaffolds hinder the process of pore formation. The right panel showcases artificially engineered PFTs (which lack the pore-forming activity) utilized as toxoid vaccines for immune cell activation. Additionally, PFTs can be biochemically manipulated for specific targeting of tumor tissues such that they remain inactive under normal conditions. Image Created in BioRender (https://BioRender.com/n76d1eb).

Taken together, these therapeutic and engineering advances underscore how a fundamental understanding of PFT biology can be harnessed for translational medicine (Figure 3).

## Future frontiers: unsolved mysteries and therapeutic potential

The comprehensive understanding of the science of PFT comes from studies focusing on the PFTs from different perspectives such as microbiology, biochemistry, biophysics, and immunobiology. However, several critical aspects of their mode of action, role in pathogenesis, and signaling pathways remain elusive. This can be attributed to the complexity of PFT mechanisms, which becomes more intricate when considered in the context of host–pathogen interactions. For each step of PFT, a cascade of molecular events can be triggered in the target cell. In a genome-wide RNA interference screen, 106 genes (∼0.5% of the genome) were identified in *C. elegans* that were involved in the protection from PFT attack [156]. This reflects the sophistication of PFT biology and the extent of biological cellular struggle between host and pathogen defenses. The first point of contact between a PFT and the target cell is mediated by the specific lipids or surface receptors. For most of the studies, lipid bilayers have been used as the platform to facilitate the pore formation. There is a large gap in the understanding of how lipid composition or composition-dependent biophysical properties of lipid bilayers can affect the binding and the pore formation. There are many PFTs for which host receptors remain unidentified, leaving the elucidation of signaling pathways and cellular tropism incomplete. Furthermore, the structural elements mediating lipid and receptor interactions require deeper dissection across toxin families. Following binding, PFTs undergo oligomerization on the membrane surface, which is believed to be mostly triggered by the increased local concentrations of toxin. However, the precise molecular cues and detailed intra- and intermolecular interactions driving this complex process remain underexplored for most of the PFTs. Further, the cellular responses to PFTs depend on whether the exposure is at lytic or sublytic concentrations. These thresholds may vary depending on both the type of PFT molecule and the target cell type. For example, immune cells are more likely to get exposed to lytic doses of PFTs, whereas non-immune cells are more likely to encounter sublytic concentrations to protect the replicative niche of the pathogen. It is intriguing how the pathogen is able to fine-tune the effective dose of PFTs according to the type of host cell it encounters. Understanding such dose-dependent and cell-type-specific interactions at the molecular level can be crucial to understanding the PFT-driven host–pathogen dynamics. In addition to the ongoing quest to understand the structural and functional roles of each PFT, their ability to affect the adaptive immune system and the possibility of transcriptional reprogramming remain speculative for most PFTs. Altogether, these questions define a dynamic and evolving frontier in PFT research. A comprehensive understanding of these mechanisms will fill the knowledge gaps in the field of PFT biology. It also holds therapeutic potential, from designing toxin inhibitors to modifying PFTs for vaccine delivery, immune modulation, or targeted cancer therapy.

## Abbreviations

AFM: atomic force microscopy
AIM2: absent in melanoma 2
ALO: anthrolysin O
CDC: cholesterol-dependent cytolysin
ClyA: cytolysin A
Cryo-EM: cryo-electron microscopy
Hla: alpha-hemolysin
ILY: intermedilysin
LLO: listeriolysin O
MACPF: membrane attack complex/perforin
MakA: motility-associated killing factor A
MAPK: mitogen-activated protein kinase
MD: molecular dynamics
NLRP3: NOD-like receptor family pyrin domain-containing 3
PAMP: pathogen-associated molecular pattern
PFT: pore-forming toxin
PLY: pneumolysin
PRR: pattern-recognition receptor
PVL: Panton-Valentine leukocidin
ROS: reactive oxygen species
RTX: repeats-in-toxin
SLO: streptolysin O
smFRET: single-molecule Förster resonance energy transfer
TDH: thermostable direct hemolysin
TLR: toll-like receptor
UPR: unfolded protein response
VacA: vacuolating cytotoxin A
VCC: *Vibrio cholerae* cytolysin
VVH: *Vibrio vulnificus* hemolysin
